# Profiling development of abdominal organs in the pig

**DOI:** 10.1038/s41598-022-19960-5

**Published:** 2022-09-28

**Authors:** George C. Gabriel, William A. Devine, Bethany K. Redel, Kristin M. Whitworth, Melissa Samuel, Lee D. Spate, Raissa F. Cecil, Randall S. Prather, Yijen L. Wu, Kevin D. Wells, Cecilia W. Lo

**Affiliations:** 1 Department of Developmental Biology, University of Pittsburgh School of Medicine, Pittsburgh, PA USA; 2 Division of Animal Sciences, Animal Science Research Center, National Swine Resource and Research Center, University of Missouri, Columbia, MO USA; 3 United States Department of Agriculture-Agriculture Research Service, Plant Genetics Research Unit, Columbia, MO USA; 4 Present Address: Department of Neuroscience, Spinal Cord and Brain Injury Research Center, and Ambystoma Genetic Stock Center, University of Kentucky, Lexington, KY USA

**Keywords:** Model vertebrates, Gastrointestinal system, Kidney, Urinary tract, Animal disease models, Gastrointestinal models, Genetic models

## Abstract

The pig is an ideal model system for studying human development and disease due to its similarities to human anatomy, physiology, size, and genome. Further, advances in CRISPR gene editing have made genetically engineered pigs viable models for the study of human pathologies and congenital anomalies. However, a detailed atlas illustrating pig development is necessary for identifying and modeling developmental defects. Here we describe normal development of the pig abdominal system and show examples of congenital defects that can arise in CRISPR gene edited *SAP130* mutant pigs. Normal pigs at different gestational ages from day 20 (D20) to term were examined and the configuration of the abdominal organs was studied using 3D histological reconstructions with episcopic confocal microscopy, magnetic resonance imaging (MRI) and necropsy. This revealed prominent mesonephros, a transient embryonic organ present only during embryogenesis, at D20, while the developing metanephros that will form the permanent kidney are noted at D26. By D64 the mesonephroi are absent and only the metanephroi remain. The formation of the liver and pancreas was observed by D20 and complete by D30 and D35 respectively. The spleen and adrenal glands are first identified at D26 and completed by D42. The developing bowel and the gonads are identified at D20. The bowel appears completely rotated by D42, and testes in the male were descended at D64. This atlas and the methods used are excellent tools for identifying developmental pathologies of the abdominal organs in the pig at different stages of development.

## Introduction

The pig is an excellent animal model for the study of disease in humans because of their anatomic and physiologic resemblances and are commonly used for research of cardiovascular, infectious diseases, metabolic, and neurological disorders in addition to developing and refining surgical and anesthetic procedures^1^. Furthermore, pigs are being genetically engineered to use as models for human diseases that include cancers, cystic fibrosis, polycystic kidney disease, diabetes, and cardiovascular diseases^2,3^. With advances in CRISPR gene editing, animal modeling, especially in the mouse, has been extremely important for investigating human diseases including congenital malformations, but mice are less morphologically and physiologically similar to humans than the pig, and CRISPR gene editing has promoted the use of genetically engineered pigs as models for the study of human diseases^4,5^. Having pig models for pathologies, including congenital malformations of the abdominal organs would be very helpful, but requires a baseline knowledge of normal development of the abdominal organs in the pig embryo/fetus. To our knowledge, using a combination of 3D reconstructions with episcopic confocal microscopy, MRI, and gross dissection of the fetal pig of early and late-stage fetuses as well as term and newborn piglets we have generated the first atlas of development of the abdominal viscera of the pig.

This publication is a companion to a previously published atlas that addressed cardiovascular development and congenital heart disease in the pig using these same imaging modalities^6^. Some of the most complex types of congenital malformations affect development of multiple organ systems including both the heart and abdominal organs. For example, heterotaxia, a severe defect in left–right patterning, can include congenital heart defects such as right or left isomerism of the atrial appendages of the heart as well as congenital malformations of the spleen, gut, kidney, and stomach. Right and left isomerism of the atrial appendages of the heart are also known as the cardiosplenic syndromes of asplenia and polysplenia, respectively, because of their associated malformations of asplenia, absence of the spleen, or polysplenia, multiple spleens, as well as other defects in symmetry such as a symmetrical or mirror image (inverted) liver, abnormal position of the gallbladder, malrotation of the bowel, abnormal position of the stomach, short pancreas, preduodenal portal vein, total anomalous venous return to the portal vein, or interruption of the inferior caval vein with azygous or hemiazygous venous return^7–9^. Identifying these abdominal organ anomalies would alert an investigator to the possibility of congenital heart disease and atrial isomerism of the appendages, or syndromic extrahepatic biliary atresia in the fetal pig. In addition, identifying a mirror image arrangement of the abdominal organs (situs inversus) may alert the researcher to the possibility of primary ciliary dyskinesia (PCD), also known as Kartagener's syndrome, which has previously been described in pigs^10^. Furthermore, other pathologies in the abdominal organs of the pig such as polycystic kidney disease, nephronophthisis, and polycystic liver disease may also alert the investigator to ciliopathies^11^.

## Results

The abdominal/pelvic organs of normal pigs at 20, 26, 30, 35, 42, and 64 days of gestational age were examined by episcopic confocal microscopy (ECM), which generates a 3D reconstruction from serially sectioned histological confocal images, and/or magnetic resonance imaging (MRI). Necropsies were performed at day 42 (D42), D64, D90, D105, term (D115), and 2-day old normal pigs as well as term piglets CRISPR edited to carry a mutation in the gene *SAP130,* encoding Sin3a associated protein 130 (Supplemental Fig. 1). The development of the following organs was evaluated: kidney, liver with gallbladder, pancreas, spleen, gastrointestinal tract, and gonads. Supplemental videos of ECM and MRI studies of embryonic and fetal pigs ranging from D20–D42 as well as the newborn *SAP130* mutant pig are included (Supplemental Videos 1–14).

**Figure 1:**
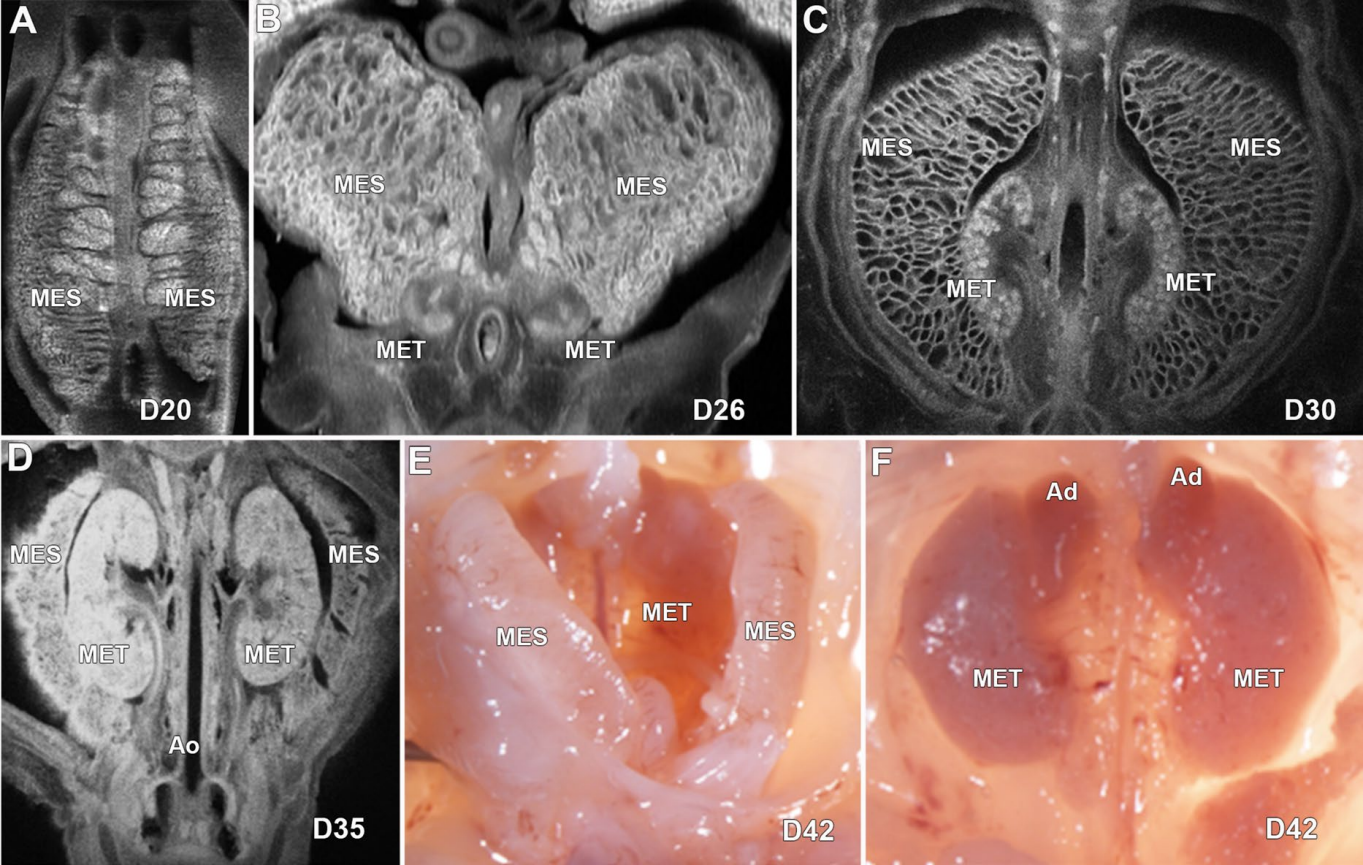
ECM and gross images of development of the kidneys in the pig from D20 to D115. Image(**A**) shows a coronal view of the mesonephric kidneys in a D20 fetus. (**B**) shows the mesonephric kidneys and the developing metanephric kidneys at D26. Images (**C** and **D**) are coronal views showing the developing metanephric kidneys at D30 and D35 respectively. Gross image (**E**) shows the mesonephric and metanephric kidneys at D42 and (**F**) illustrates the kidneys with adrenal glands after the mesonephric kidneys have been removed. MES-Mesonephros, MET-Metanephrous, Ao-Aorta, Ad-adrenal gland.

### Kidneys

At D20 the mesonephroi were well-developed and the metanephros kidneys were not grossly identified (Fig. 1A, Supplemental Fig. 2A). By D26, early metanephric kidneys are identified (Fig. 1B), and at D30 the metanephric kidneys have developed their characteristic shape and their parenchyma have a weakly consolidated appearance without obvious cortical-medullar junctions, and with prominent calyces and pelvi and discernible ureters (Fig. 1C). At D35, the parenchyma of the kidneys has a more consolidated appearance, and the cortical-medullar junctions are weakly recognizable (Fig. 1D). Gross dissection of the abdomen at D42 shows well-formed mesonephroi, and externally normal appearing kidneys and adrenal glands (Fig. 1E,F). At D64, the kidneys are completely formed and the mesonephroi were no longer identified. Further gross analysis of the 2-day old kidney shows the kidney location within the abdominal cavity and dissection of the kidney revealed the renal cortex, renal medulla, renal papilla, calyces, and renal pelvis (Supplemental Fig. 2B,C).

**Figure 2:**
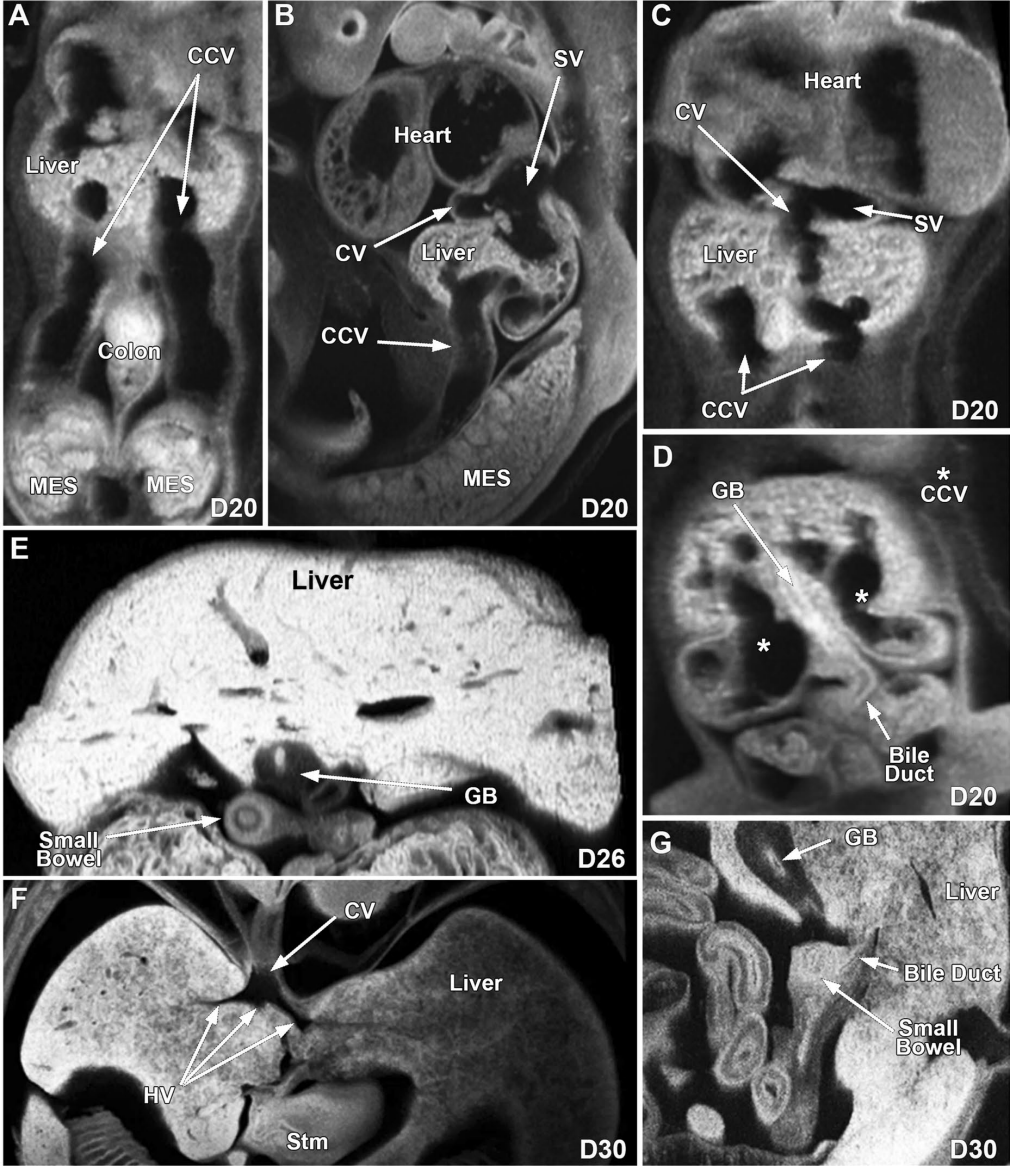
ECM imaging of developing caval veins, gallbladder, and liver in the pig at D20, D26 and D30. (**A**) shows the developing liver and caudal cardinal veins, (**B** and **C**) shows the supra-hepatic portion of the caval vein, sinus venosus and developing liver, and (**D**) shows the developing liver, gallbladder, and bile duct at D20. Image (**E**) shows the developing liver and gallbladder at D26. Illustration (**F**) shows the supra-hepatic caval vein and hepatic veins, and (**G**) shows the gallbladder with the bile duct connected to the small bowel at D30. CCV-Caudal Cardinal vein, SV-Sinus venosus, CV-Cava vein, GB-Gallbladder, MES-Mesonephros, Stm-Stomach, HV-Hepatic vein.

### Liver and gallbladder

**Figure 3:**
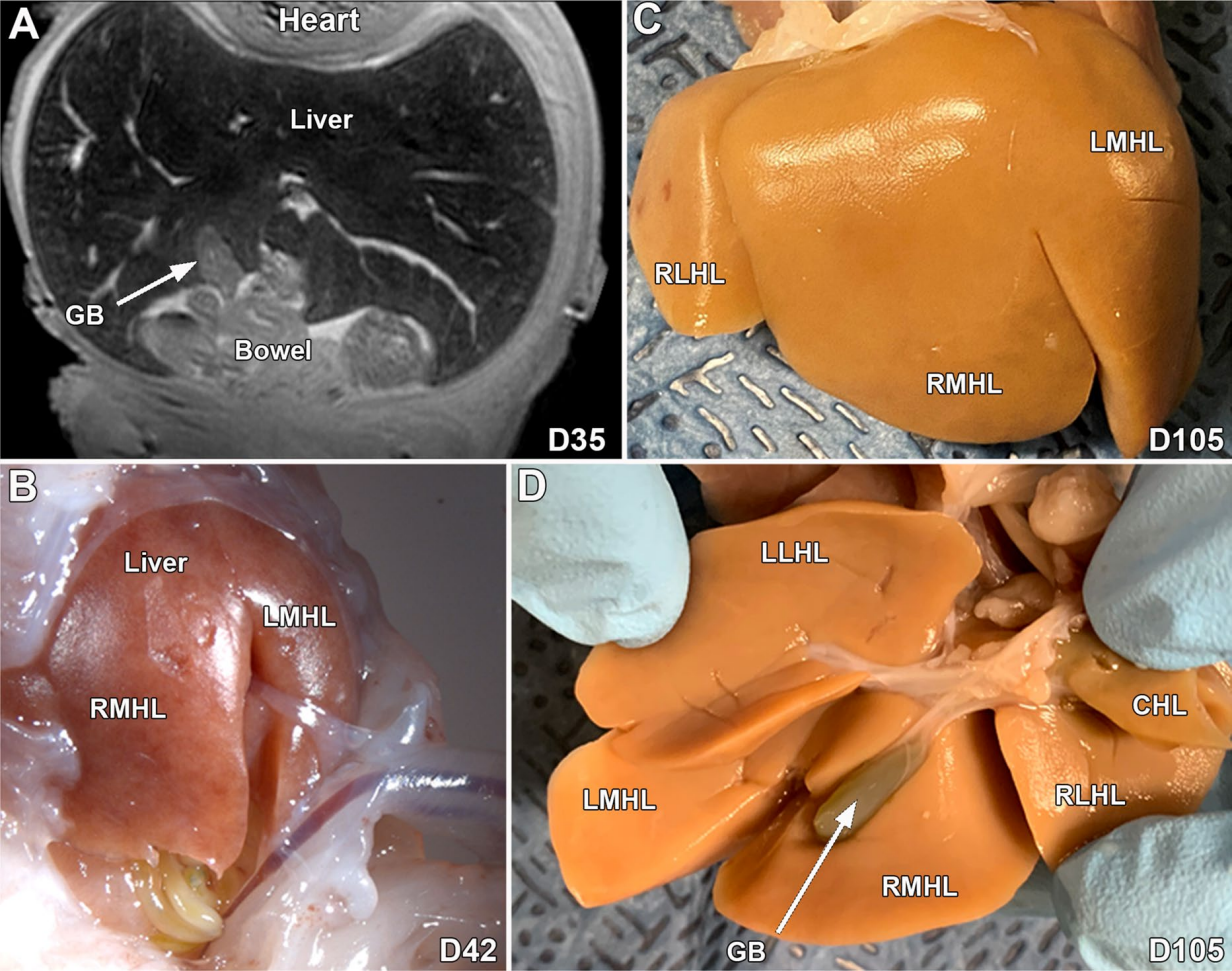
MRI and gross images of the developing liver in the pig at D35, D42 and D105. An MRI image at D35 (**A**) shows the well-developed liver and gallbladder, (**B**) is an in situs gross image of the liver and gallbladder at D42. Gross images (**C** and **D**) show the well-developed liver and gallbladder at D105. CHL- Caudate hepatic lobe, GB-Gallbladder, RLHL-Right lateral hepatic lobe, RMHL-Right medial hepatic lobe, LMHL-Left medial hepatic lobe, LLHL-Left lateral hepatic lobe.

At D20, the liver is small and has a weakly consolidated appearance with easily recognized bilateral caudal cardinal veins, and identifiable supra-hepatic caval vein and sinus venosus (Fig. 2A–C). In addition, a small gallbladder and bile duct can be seen (Fig. 2D). At D26, the liver appears more amalgamated, the gallbladder is thick walled and the inferior caval vein is present with absence of one of the caudal cardinal veins (Fig. 2E). At D30, a larger, well-formed liver with a more consolidated appearance and well delineated caval, hepatic and portal veins, bile ducts and a gallbladder that continues to have a thick wall (Fig. 2F,G, Supplemental Fig. 3A,B). Images at D35 show a more compact liver (Fig. 3A). Necropsy of the D42 fetal pig shows a well-formed liver of appropriate size and lobation (Fig. 3B), and similarly at D105, five distinct lobes and a normal appearing gallbladder are observed (Fig. 3C,D). The common bile duct, hepatic bile ducts, cystic bile duct, and gallbladder can be identified in the 2-day old pig (Supplemental Fig. 3C).

### Pancreas

**Figure 4:**
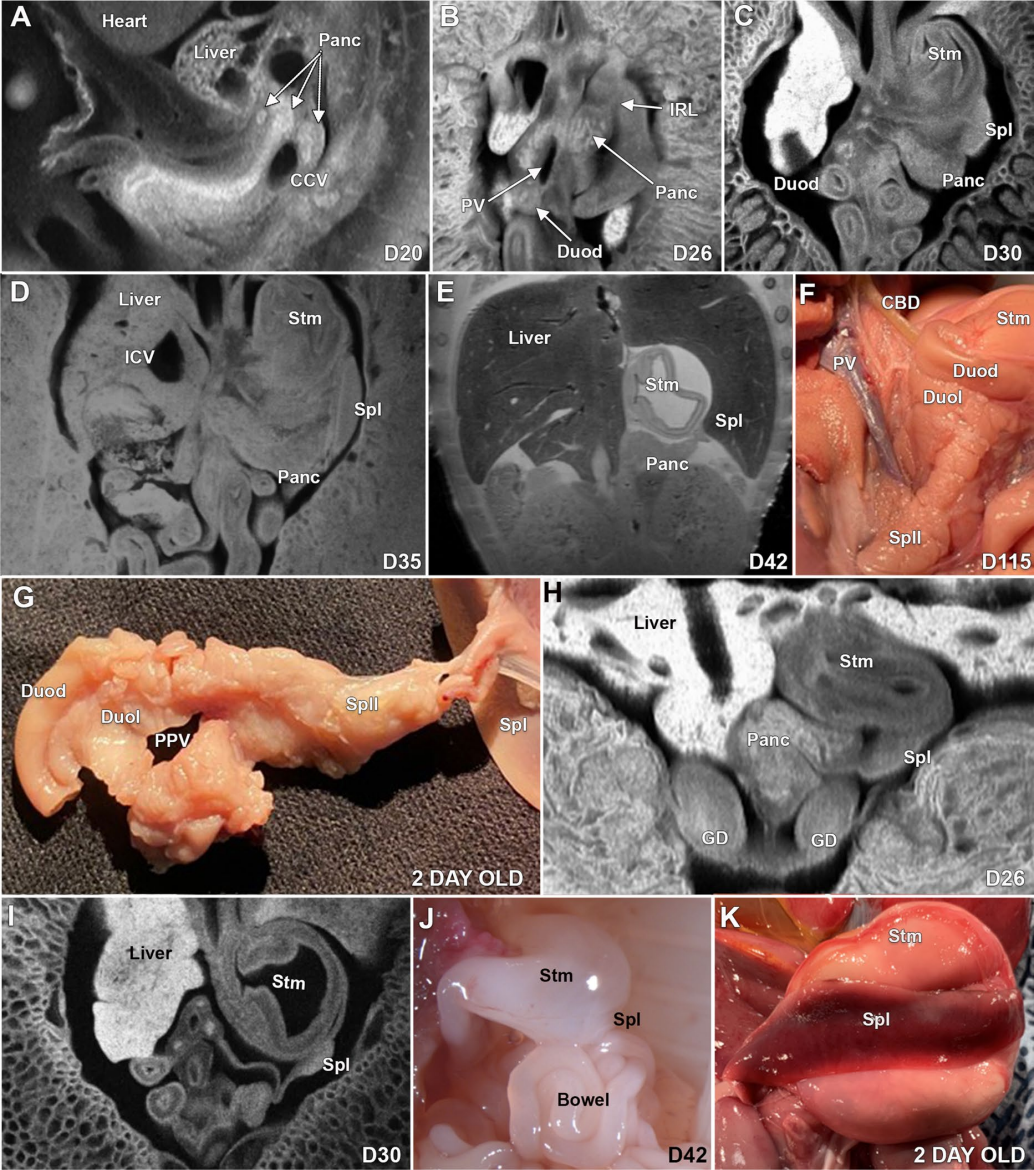
ECM, MRI, and gross images of the developing pancreas and spleen in the pig at D20 to 2 days of age. The early development of the pancreas (**A**) is seen at D20, and at D26. Image (**B**) shows the pancreas and its relationship to the portal vein. Image (**C**) shows the tail of the pancreas extending to the spleen at D30, and at D35, the pancreas appears more consolidated and well-formed (**D**), and at D42, the pancreas appears completely grossly formed (**E**). The in-situ dissection shows the pancreas surrounding the portal vein (**F**), and (**G**) shows the ex-situ dissected pancreas of a 2-day old pig. Images (**H** and **I**) show the spleen attached to the stomach at D26 and D30 respectively. Illustration (J) is an in-situ image of the pale spleen at D42 and (**K**) shows an in-situ spleen at 2 days of age. Panc-Pancreas, CCV-Caudal cardinal vein, PV-Portal vein, Duod-Duodenum, IRL-Ilenorenal ligament, ICV-Inferior caval vein, Stm-Stomach, CBD-Common bile duct, DuoL- Duodenal pancreatic lobe, Spll-Splenic pancreatic lobe, PPV-Path of the portal vein, GD-Gonads.

At D20, ECM imaging shows lobulated pancreatic tissue that is associated with a caudal cardinal vein (Fig. 4A). At D26, the pancreas is surrounding the portal vein, the pancreatic tail extends towards the spleen and the pancreatic lobules are prominent and not consolidated (Fig. 4B). By D30, the pancreas is well-formed and more consolidated (Fig. 4C, Supplemental Fig. 4A–D), progressing to being almost completely consolidated by D35 (Fig. 4D) and fully consolidated by D42 (Fig. 4E). At D64, the pancreas is well-formed, and the tail of the pancreas extends to the spleen. The in-situ dissection of the pancreas at D115 is shown in Fig. 4F, and the ex-situ pancreas of a 2-day old is shown in Fig. 4G.

### Spleen

At D20, the spleen was not positively identified but the spleen was easily identified at D26 and D30 (Fig. 4H,I). In situ dissection at D42 showed a small, pale spleen (Fig. 4J) and the spleen at 2 days of age had the normal red coloration and size (Fig. 4K).

### Gastrointestinal tract

**Figure 5:**
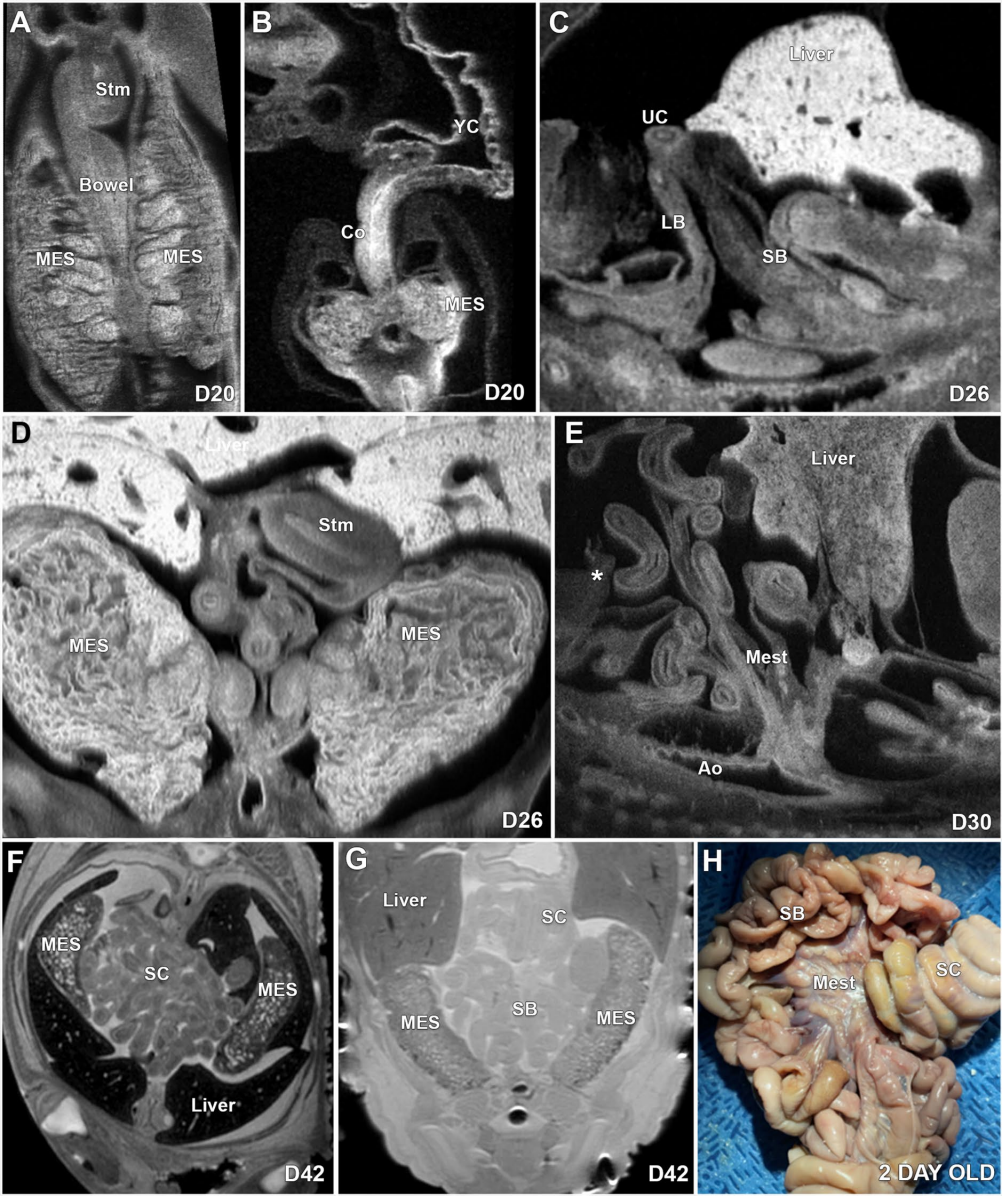
ECM, MRI and gross images of the development of the stomach and intestine in the pig at D20 to 2 days of age. (**A**) shows the early developing stomach and (**B**) shows the relationship of the colon to the yolk sac at D20, and at D26, bowel has herniated to the base of the umbilical cord with part of the bowel located outside the abdominal cavity (**C**) and the bowel is in the process of rotating (**D**). At D30 (**E**), a portion of the bowel is still herniated through the normal opening in the fetal abdominal wall and the * shows the posterior limit of the abdominal wall opening and the mesentery is well-formed. At D42, the bowel is completely located within the abdomen, the abdominal wall is intact, and the rotation of the bowel appears completed (**F**) and by MRI, the spiral colon is well-formed (**G**). Illustration (**H**) is an ex-situ specimen of the small bowel and spiral colon in a 2-day old pig. Stm-Stomach, MES-Mesonephros, YS-Yolk sac, Co-Colon, UC-umbilical cord, SB-Small bowel, LB-Large bowel, Mest-Mesentery, Ao-Aorta, SC-Spiral colon, *- Border of the fetal abdominal wall.

The stomach at D20 is seen as a left-sided pouch with a small lumen in the expected region of the abdomen (Fig. 5A), and at D26 the stomach shows rotation, and the gastric lumen appears larger (Fig. 5D). By D30, the stomach has the expected appearance with an obvious lumen (Fig. 4C). At D20, the intestine has elongated, and a portion is herniated through the normal abdominal wall opening and bowel rotation is not appreciated (Fig. 5A,B). By D26, additional bowel has herniated through the normal abdominal wall opening (Fig. 5C), and the elongation and rotation of the bowel are underway (5D). At D30, a portion of the bowel still herniated through the normal opening in the fetal abdominal wall, and the mesentery is well-formed and with continuing rotation of the bowel (Fig. 5E). MRI scans of the D42 fetus shows retraction of the bowel complete, with the entire bowel located within the abdomen (Fig. 5F,G), An intact abdominal wall is present, and rotation of the bowel appears complete with the spiral colon well-formed (Fig. 5F,G). An ex-situ gross intestinal specimen of a 2-day old pig shows a well-formed mesentery, small bowel, and the spiral colon (Fig. 5H).

### Adrenal glands

**Figure 6:**
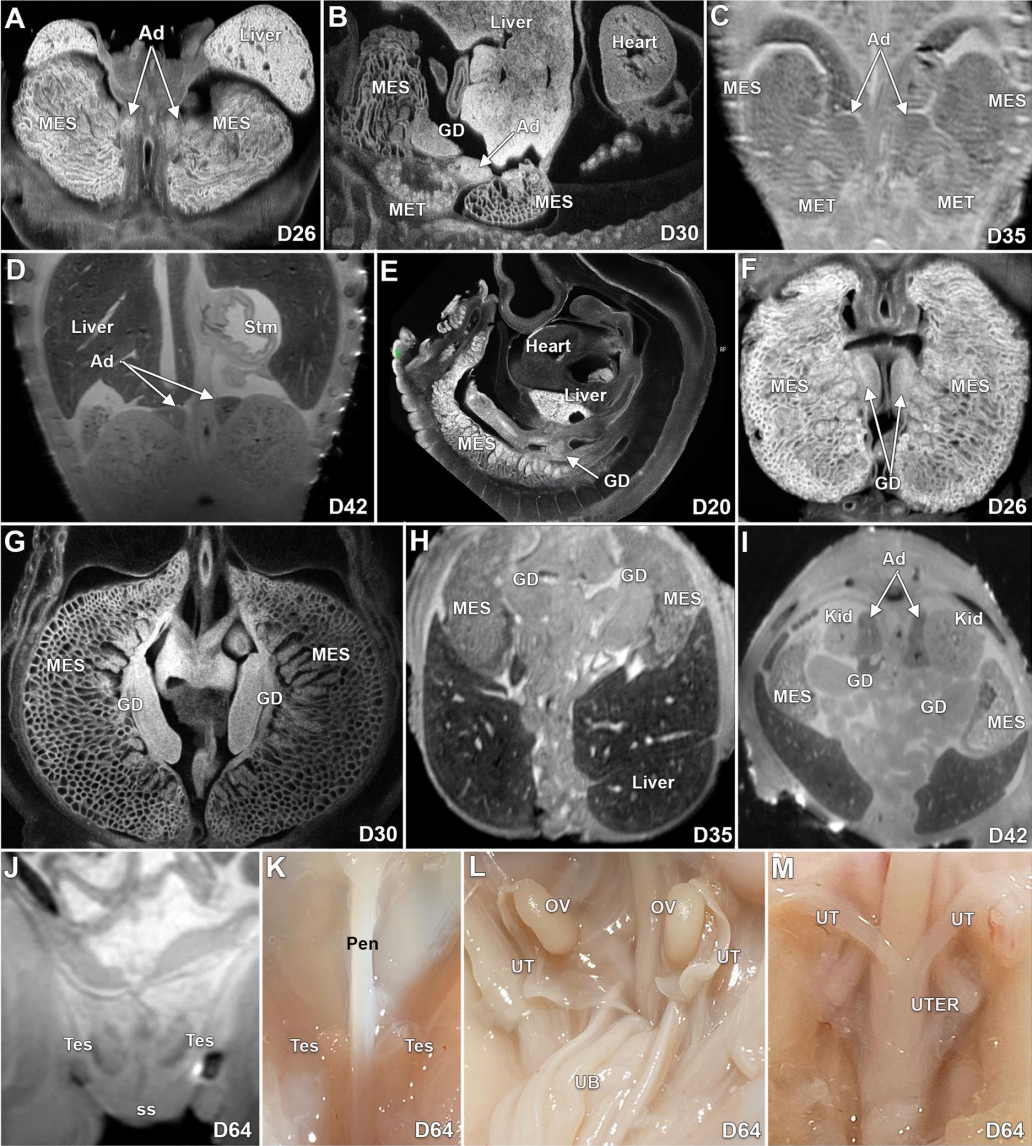
ECM, MRI and gross images of the development of the adrenal glands and gonads in the pig from D20 to D64. Adrenal tissue was first identified at D26 days (**A**) and can be easily identified at the upper poles of the kidneys at D30 (**B**) and D35 (**C**). At D42, the adrenal glands appear well-formed, and their cortex and fetal cortex can be identified (D). Possible gonadal ridges were identified at D20 near the liver (**E**). Between D26 and D30 the gonads appear to elongate in the caudal direction (**F** and **G**). At D35 and D42 the gonads are still located within the abdomen (**H**, **I**). By D64 the testes have migrated to the scrotal area as seen by MRI (**J**) and gross dissection (**K**), and the ovaries and uterine tubes (**L**) and the uterus (**M**) are also identified by gross dissection. Ad-adrenal glands, MES-Mesonephros, Kid-Kidney, MET-Metanephros, Stm-Stomach, GD-Gonads, Tes-Testis, ss-Scrotal sac, Pen-Penis, OV-Ovary, UT-uterine tube, UB-Urinary bladder, UTER-Uterus.

We first identified the developing adrenal glands at D26 (Fig. 6A); they were not associated with the metanephroi. At D30, the adrenal glands appeared attached to the upper poles of the small developing kidneys (Fig. 6B). The adrenal glands became more consolidated and increased in size by D35 (Fig. 6C). At D42, the adrenal glands are well-formed and consolidated with an adrenal cortex and a central area (fetal cortex) destined to develop into the adrenal medulla (Fig. 6D).

### Gonads

Possible gonadal ridges (not confirmed by histology) can be seen at D20 (6E). They appear to elongate in the caudal direction and descend caudally as development progressed. Between D26-D35, the gonads are elongated, have an ovoid configuration and are located medial and at the level of the midportion of the mesonephroi (Fig. 6F–H). At D42, the gonads are located lower (posterior in the pig) in the abdominal cavity and positioned at the level of the kidneys (Fig. 6I). At D64 the testes appear descended in the male (Fig. 6J,K), and, the ovaries, uterine tubes, and uterus are well formed in the female (Fig. 6L,M).

### *SAP130* mutant pig

**Figure 7:**
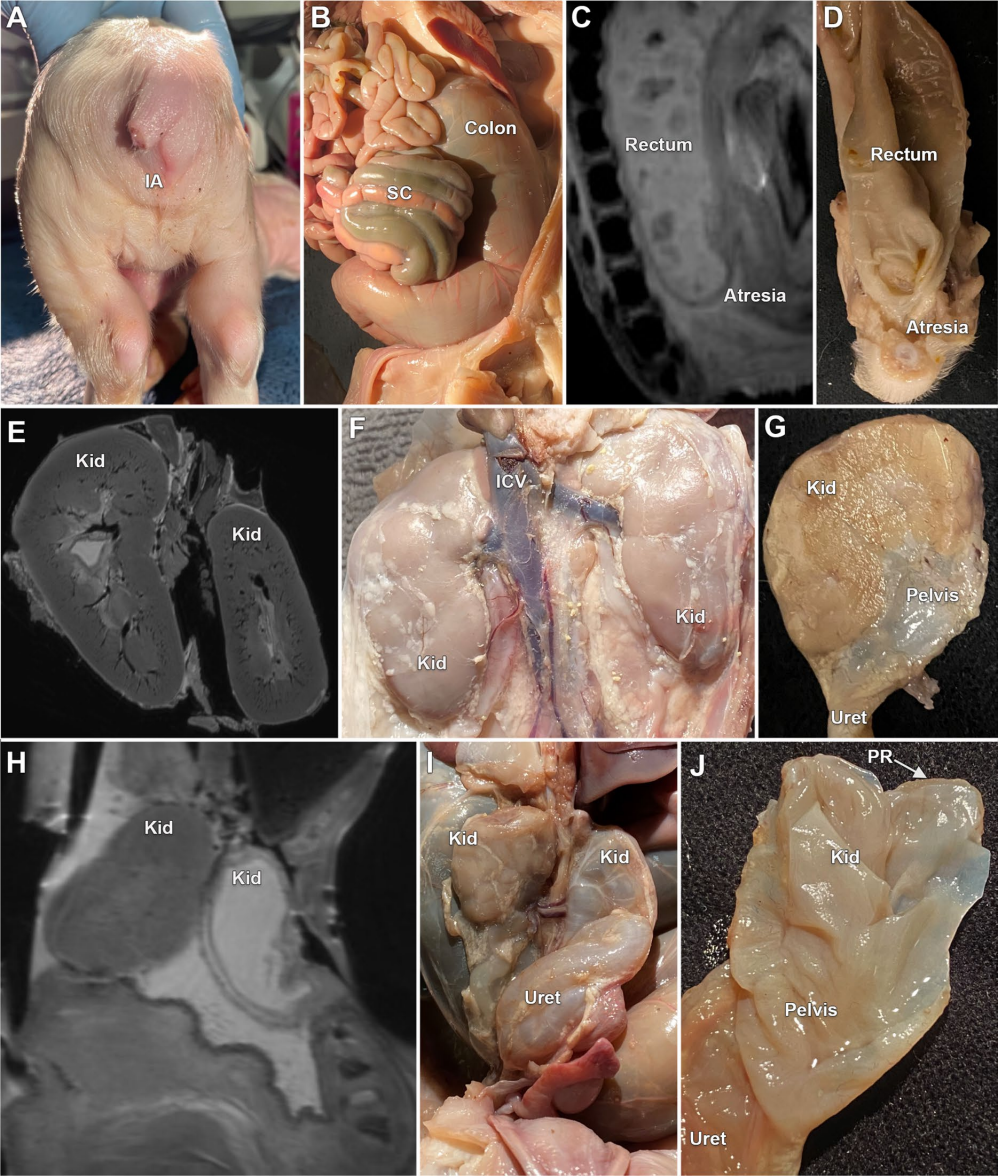
MRI and gross images of the malformations identified in a mutant *SAP130* term pig. Image (**A**) shows the anal region showing the imperforate anus. Most of the tail has been removed. The in situ gross image of the abdomen shows the distended colon (**B**). A sagittal MRI (**C**) shows the distended colon and rectum and the site of the atresia, and (**D**) is the gross dissection of the atretic rectum. Image (**E**) is an MRI of normal (wild type) kidneys and (**F**) shows the gross appearance of normal pig kidneys. Illustration (**G**) is the cut surface of hypoplastic kidney found in the mutant pig. An MRI of the mutant pig kidneys (**H**) shows one hypoplastic kidney and one hypoplastic kidney with severe hydronephrosis with a hydroureter. Image (**I**) shows the gross appearance of the hypoplastic kidneys with the one kidney showing hydronephrosis with a hydroureter. The opened hypoplastic and hydronephrotic kidney of the mutant (**J**) showing the severe hydronephrosis with the loss of renal architecture with only a narrow rim of renal parenchyma identified. IA-Imperforate anus, SC-Spiral colon, ICV-Inferior caval vein, Kid-Kidney, Uret-Ureter, RP-Renal parenchyma.

MRI, gross examination, and dissection of a CRISPR gene-edited *SAP130* mutant term pig showed multiple malformations that consisted of an imperforated anus with rectal atresia (Fig. 7A–D), kidney defects when compared to normal age matched controls (Fig. 7E,F) including hypoplastic kidneys with one kidney showing very severe hydronephrosis with a thin rim of renal parenchyma and hydroureter and the other hypoplastic kidney showing a moderate hydroureter (Fig. 7G–J). In addition, this *SAP130* piglet showed tricuspid valvar dysplasia, an atrial septal defect, and abnormal head and facies.

## Discussion

Animal modeling of human diseases has been invaluable in advancing our understanding of disease mechanisms and for the development of evidence-based therapies. With wide-spread use of mutant and transgenic mice for modeling human diseases, several mouse developmental atlases have been created by using micro-CT or MRI to provide baseline data on normal development needed to ascertain the etiology of malformations and associated disease pathology^12–17^. With the recent advancements in gene-editing technology and somatic cloning in pigs, genetically engineered pig models have become much more accessible. As a result, pigs are ever increasing in demand for modeling human diseases, especially malformations associated with prenatal/neonatal lethality more difficult to study in the much smaller size mouse model. Hence, the construction of a developmental atlas of the pig is compelling, and to this end, we have previously established an atlas of cardiovascular development in the pig, and now a pig atlas of abdominal organ development.

Our study utilized 3D reconstructions with episcopic confocal microscopy, MRI, and also gross dissection for evaluating the gross morphology of abdominal organs in the fetal pig, including structures as small as the extrahepatic bile ducts or the mesenteric artery. Evaluation of the abdominal organs of the pig embryo and early fetus by episcopic confocal microscopy and/or MRI can reduce the need for necropsies, which is not only time consuming but also requires great skill, especially when analyzing the smaller size embryos or fetuses. Our study documenting the developmental profile of abdominal organ development in the pig will facilitate future analysis of genetically engineered pig models for investigating the etiology and disease mechanisms contributing to abdominal organ pathology.

**Figure 8:**
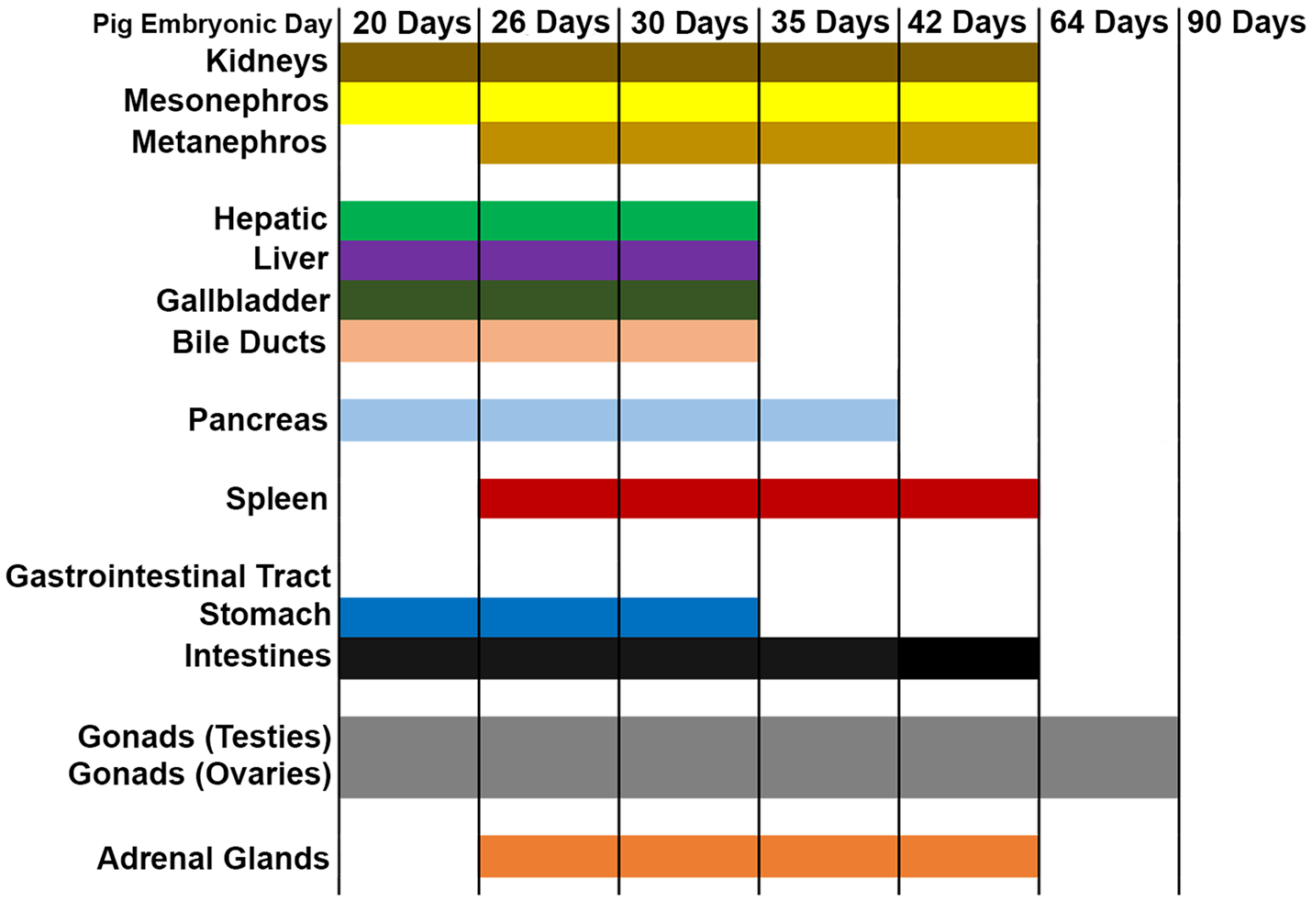
Timeline of the gross morphologic development of the abdominal organs in the pig (*Sus scrofa)*. The cells at the top of each column of the graph indicate the gestational age that the studied organs appear to have obtained their distinct shape and expected gross appearance of their parenchyma but do not indicate the final stages of development which continue to develop in size as well as at the cellular and molecular levels postnatally.

The abdominal organs of the pig have very similar anatomy to that of the human, and thus allow abdominal pathologies and malformations to be faithfully modeled^1,2^. Of the abdominal organs studied including kidney, liver, gallbladder, pancreas, spleen, gastrointestinal tract, gonads, and adrenal glands, all but two, the spleen and adrenal glands, could be identified by their gross appearance at 20 days GA. However, by 26 days GA, spleen and adrenal glands could be observed. The liver with gallbladder and bile ducts and pancreas appear to be well formed by GA 35, while the kidneys, spleen, bowel, and adrenal glands appear to have completed their formation by 64 day GA. At 64 days, the gonads also appear to be well on their way to being fully formed, with the testes migrating into the scrotum area. This temporal profile of gross development of the pig abdominal organs are summarized in Fig. 8. While this timeline tracks emergence of the organ with a gross appearance similar to that of the adult organ, we note these organs will continue to increase in size and undergo maturation at the cellular and biochemical levels. For the kidney, at 20 days GA, the mesonephroi were well developed but the metanephroi were not grossly identified until 26 days GA. By 64 days, the mesonephroi were no longer observed, while the metanephroi remain well formed. Despite external gross appearance showing fully formed kidneys at 64 days, nephrogenesis is actually not completed until 3 weeks postnatally^18,19^.

Using the *SAP130* mutant pig, we investigated for abdominal organ defects, as our previous studies in mice showed mutation in this gene can cause cardiac and extracardiac defects. Defects were observed with incomplete penetrance, similar to findings in the mutant mice^20^. Externally the *SAP130* mutant term piglet showed imperforate anus with absence of an anal opening. This was demonstrated with an MRI scan that showed a distended blind-ending rectum filled with white material, observations that also illustrated the utility of this non-invasive imaging method for evaluating congenital malformation. Also observed were renal malformations with hypoplastic kidney and hydronephrosis. We noted previously surgically produced kidney obstruction in the pig have been studied using MRI^21,22^. Pig model also would be invaluable for future modeling of malformations involving the gallbladder or the biliary ducts, such as in extrahepatic biliary atresia, a condition that is still clinically challenging to manage with no good therapeutic options. Biliary atresia is difficult to study in mice, given the small size of the biliary ducts, making a pig model compelling. A related abdominal defect, gut malrotation, also may be better modeled in pigs. These and other abdominal organ defects can be better investigated in the pig, especially those that are difficult to study in rodent models. Such studies will be greatly aided by the abdominal developmental atlas developed in this study.

Overall, the pig is an excellent animal model for the study of pathologies and malformations of the abdominal organs in human diseases. Among different larger animal models available, the pig is ideally suited for modeling human pathologies, not only given its similar anatomy and physiology, but also the pigs have a fairly short gestational period of only 114 days. Pigs also come into estrus in 26–28 days after giving birth and they have litter sizes of between 7 to 14 piglets, similar to mice. This is particularly beneficial when working with transgenic mice when specific genotypes are required to model disease. Importantly, the larger size of the fetal pig would allow smaller structures such as the hepatic bile ducts, ureters and abdominal vasculature to be more easily evaluated whether using advanced imaging such as MRI or even with gross dissection and also with episcopic confocal microscopy. With the pig abdominal atlas developed in this study, this can help streamline the use of pigs to model diseases involving the abdominal organs.

## Materials and methods

### Animals

Large white domestic crossbred pigs (Sus scrofa) were used in this study. An atlas focused on pig cardiovascular development was previously published using these same animals^6^. All animal work was humanely conducted under an approved University of Missouri IACUC protocol and according to ARRIVE guidelines. All wildtype animals studied, including both fetuses and newborns, were generated by Landrace Large White cross parent gilt with semen from Choice USA Genetics (Choice USA, West Des Moines, IA). All pigs used for this study were raised on an approved farm facility and then moved into a University of Missouri animal facility for sample collection. All facilities are approved for biomedical pigs by the University of Missouri Animal Care and Use Committee and followed the Guide for the Care and Use of Laboratory Animals and the program is AAALAC accredited. The *Sap130* mutant pig used in this study was generated previously with RRID, NSRRC:0081^6^.

### Breeding and harvesting pig specimens and in vivo fetal collections

Breeding and harvesting of fetal and newborn pigs was carried out as previously described^6^. Briefly, wild type gilts were bred by artificial insemination with wild type semen. Day 0 of gestation was classified as the first day of detectable estrus, and pregnant female pigs were humanely euthanized on day 20, 26, 30, 35, 42, 64, 90 or 115 of gestation (referred to as D20, D26, D30, D35, D42, D64, D90, D115). Specimens up to 35 GA are referred to as embryos as they are largely indistinguishable between mouse, pig, human, but those at 42 days GA and beyond are referred to as fetuses, as at these stages they have craniofacial and limb features distinct for the pig. The stages selected for the present study are based on our earlier analysis of the temporal profile of cardiovascular development in the pig, with D20 corresponding to early heart development comprising the looped heart tube developmental stage^6^. At this stage, neither ventricular, atrial, nor outflow septation has occurred. As the heart is the first organ system to form, this provided a reasonable starting point for profiling development of the abdominal organs, which are initiated after formation of the heart. From our previous study, we had determined spacing at ~ 5–6 day intervals can maximize what can be learned regarding the developmental progression of organogenesis at these early stages. However, the exact day of collection varied by a day or two, determined by availability of staff for collection of the specimen. Beyond day 35, we collected and analyzed fetuses at increasingly larger intervals that spanned Day 42, Day 64, Day 90 and Day 115 (newborn). By Day 42, most abdominal organs are fully formed, except for the gonads whose development continues at Day 64, but is completed by day 90.

For the embryo/fetus collection, the uterus was opened on the antimesometrial side and fetuses were removed. The whole fetus from each stage was then drop-fixed in 4% paraformaldehyde at room temperature. For fetuses at D42, D64 and D90, a small opening on the side of each fetus was introduced to allow fixative to permeate the chest cavity. Newborn piglets were kept on ice until dissection of all organs. Collected organs were photographed and placed in fixative. Embryos/fetuses were fixed in 4% PFA or 10% buffered formalin for 2–5 days. At minimum, three embryos/fetuses per stage were analyzed. D20 to D42 embryos/fetuses were necropsied by using a stereomicroscope with digital images captured using the Kontron Progres digital camera. MRI scans were conducted followed by histological reconstructions using episcopic confocal microscopy. Newborn pigs (D115) and 2-day old pigs were analyzed by gross dissections and individual organs were separated and further analyzed by MRI. All animal work was humanely conducted under an approved University of Missouri IACUC Protocol.

### Generation of *SAP130* mutant pigs

The *SAP130* mutant piglet was generated by a het x het mating of male 24–3 and female 24–4. The male (24–3) contains a 7 bp deletion and the female (24–4) contains a 4 bp deletion in *SAP130*. Six piglets were born. The piglets were genotyped in the same manner as Gabriel et al.^6^. After the piglets were genotyped, it was identified that one of the founder pigs was mosaic and contained a second modified allele, a 6 bp deletion with 2 bp mutation. It is not clear, which founder pig contained the third allele. Only one *SAP130* mutant piglet was born from this litter containing an allele with a 4 bp deletion and an allele with a 7 bp deletion which resulted in a *SAP130* null genotype. The mutant piglet was identified by external phenotype and euthanized for analysis at term.

### Histopathology with episcopic confocal microscopy

For embryos at D20, D26, D30, and D35, following necropsy and MRI, the whole fetus or only the abdominal section of the fetus was embedded in paraffin for episcopic confocal microscopy (ECM). Paraffin embedded samples were sectioned using a Leica SM2500 sledge microtome and serial confocal images of the block face were captured using a Leica LSI scanning confocal macroscope mounted above the sample block as previously described^6^. The 2D serial image stacks collected were visualized using the OsiriX Dicom viewer11 (https://www.osirix-viewer.com). These image stacks could be digitally re-sectioned in multiple imaging planes and 3D reconstructed for optimal viewing of the abdominal organs.

### Magnetic resonance imaging

Prior to MRI scanning, embryos/fetuses were fixed and stained with a gadolinium (Gd)-based contrast agent to shorten the tissue T1. Briefly, after fixation embryos/fetuses were immersed in 1:200 MultiHance^23^ (gadobenate dimeglumine, 529 mg/ml, Bracco Diagnostic, Inc. Monroe Twp, NJ) diluted with phosphate-buffered saline (PBS) at 4^0^C for at least 48 h. After staining, small embryos were secured on a tongue depressor (McKesson Medical-Surgical, Irving, TX) with Webglue surgical adhesive (n-butyl cyanoacrylate, Patterson Veterinary, Devens, MA). The embryos/fetuses were then immersed in Fomblin Y (perfluoropolyether, Sigma-Aldrich Millipore) to eliminate the susceptibility artifact at the tissue-air interface and to avoid dehydration during imaging.

MRI was carried out as previously described, with special emphases on abdominal structures, using a Bruker Biospec 7 T/30 system (Bruker Biospin MRI, Billerica,MA) with a 35-mm or 72-mm quadrature coil for both transmission and reception^6^. 3D MRI was acquired with a fast spin echo sequence, the Rapid Acquisition with Refocusing Echoes (RARE), with the following parameters: effective echo time (TE) 24.69 ms,RARE factor 8, repetition time (TR) 900 ms. We used RARE also known as Fast Spin-Echo (FSE) or Turbo Spin-Echo (TSE) pulse sequence for high-resolution 3D imaging with T_2_-weighted contrast. It generates similar T_2_-weighted contrast as the Half-Fourier-Acquired Single-shot Turbo spin Echo (HASTE), a Turbo spin-echo technique that is used for sequential acquisition of high-resolution T_2_-weighted images. However, the strategy for fast spin echo is different. Our RARE condition with RARE factor 8 uses 8 echoes as 8 phase-encoded k-space lines to accelerate acquisition; whereas HASTE uses a single-shot technique or segmented multiple shorts to cover sufficient k-space from a single TR. HASTE although commonly used in human scanners, it is not available in the Bruker preclinical scanner used in this study. RARE provides flexible T_2_-weighting conditions by changing RARE factors depending on the tissue types of interest. We have tested various RARE factors, TE, and TR combinations to optimize the contrast, signal to noise ratio (SNR), and scan time used in this study.

The field of view (FOV), acquisition matrix and voxel sizes varied based on the sample size. The typical spatial resolution for D26, D30, and D35 embryos ranged from 39 µm to 46 µm, that of D42, D64 and D90 fetuses ranged from 45 µm to 62 µm. The FOV, matrix, resolution, echo time, RARE factor, and other MR parameters used for imaging at the different GAs are provided in Supplemental Spread Sheet 1. The 3D MRI imaging stacks were exported with DICOM format and could be re-oriented to any viewing angle with Horos Dicom Viewer (Horosproject.org).

### Gross examination

Necropsies were performed as previously described on D42, D64, D90, D105, D115 and 2-day old wildtype normal pigs which showed no external malformations^6^. Briefly the thoracic, abdominal, and pelvic viscera were examined in situ for malformations, the heart, great vessels, and lungs were removed as a block and examined using the sequential segmental analytical method^24,25^. Following examination of thoracic organs, the abdominal-pelvic visceral blocks were removed as a block and dissected and examined from behind (dorsal in the pig). Because pigs are quadrupeds, structures, which in bipedal mammals are described as inferior in pigs are described as posterior or caudal, for example the inferior caval vein can be referred to as the posterior or caudal caval vein in the pig. However, to better align the pig to the bipedal mammal, we have chosen, like others, to describe the abdominal organs of pigs as in bipedal mammals^26^. The abdominal organs in mammals obtain their basic gross appearance before term but continue to develop after birth by increasing in size or length as well as at the cellular and biochemical levels. In addition, in the very early embryo the organs begin by cell differentiation, and they lack the expected configuration that is seen in the fetus. In this study we focus on the assessment of the basic gross appearance of the organs before term.

## Supplementary Information

Supplementary Information 1.

Supplementary Video 1.

Supplementary Video 2.

Supplementary Video 3.

Supplementary Video 4.

Supplementary Video 5.

Supplementary Video 6.

Supplementary Video 7.

Supplementary Video 8.

Supplementary Video 9.

Supplementary Video 10.

Supplementary Video 11.

Supplementary Video 12.

Supplementary Video 13.

Supplementary Video 14.

Supplementary Information 2.

## Data Availability

All data generated or analyzed during this study are included in this published article (and its Supplementary Information files).
