# Supplementary material for: Profiling development of abdominal organs in the pig
Source: Sci Rep. 2022 Sep 28;12:16245. doi: 10.1038/s41598-022-19960-5 (PMC9519580; doi:10.1038/s41598-022-19960-5)
Supplement: Supplementary file 16 — Supplementary Information 2. [file 41598_2022_19960_MOESM16_ESM.pdf]

SUPPLEMENTAL DATA

Supplemental Table 1. A comparison between mouse and pig (*Sus scrofa)* of the developing abdominal organs when they obtained their distinct shape and expected gross appearance of their parenchyma. The final stages of development which continue postnatally at the cellular and molecular levels were not studied.

Mouse Development (days)	Pig Development (days)	Morphogenetic Event
**E9.0-E15.5**[1,2]	20-26	Liver, gallbladder and bile duct formation
**E9.5 (Primitive gut tube)**	20-42	Bowel Formation
**E9.5-E16**	20-35	Pancreas Formation
**E8.5-E12-13 (Range 12.5 - 14)**[3-5]	20-64	Kidneys
**E8.5-E14/15 (Range 13.5 - 15)**	20-64	Mesonephroi
**E10.5-E12-13 (Range 12.5 - 14)**	26-64	Metanephroi
**E10-E13**[6]	20-64	Gonads
**E10.5/11-12.5**	26-35	Spleen Formation
**E9-E14.5**[7]	26-42	Adrenal Gland Formation

SUPPLEMENTAL FIGURES

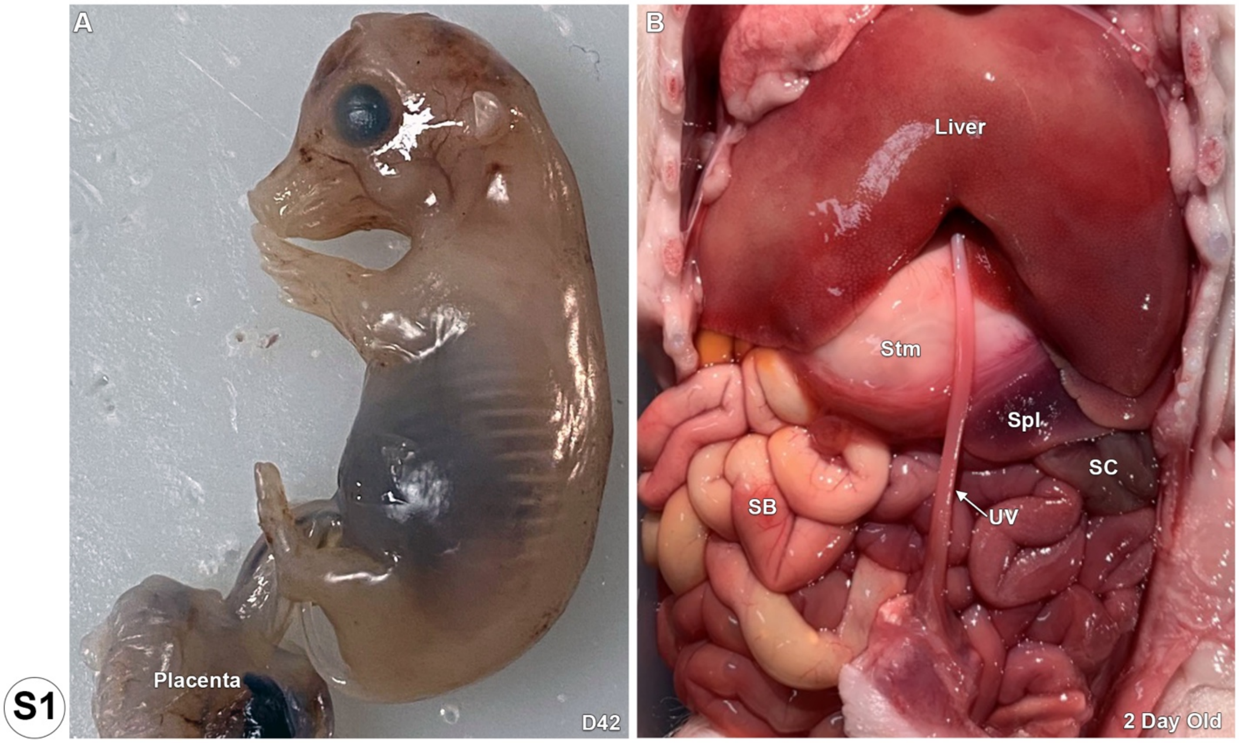

Supplemental Figure 1. Images of a fetal pig and postnatal in-situ abdominal organs of a 2-day old pig. A sagittal view of a D42 GA pig fetus is displayed in panel (A) and an in-situ view of the abdomen of a 2-day old pig is shown in (B). Stm-Stomach, Spl-Spleen, SC-Spiral colon, UV-Umbilical vein, SB-Small bowel.

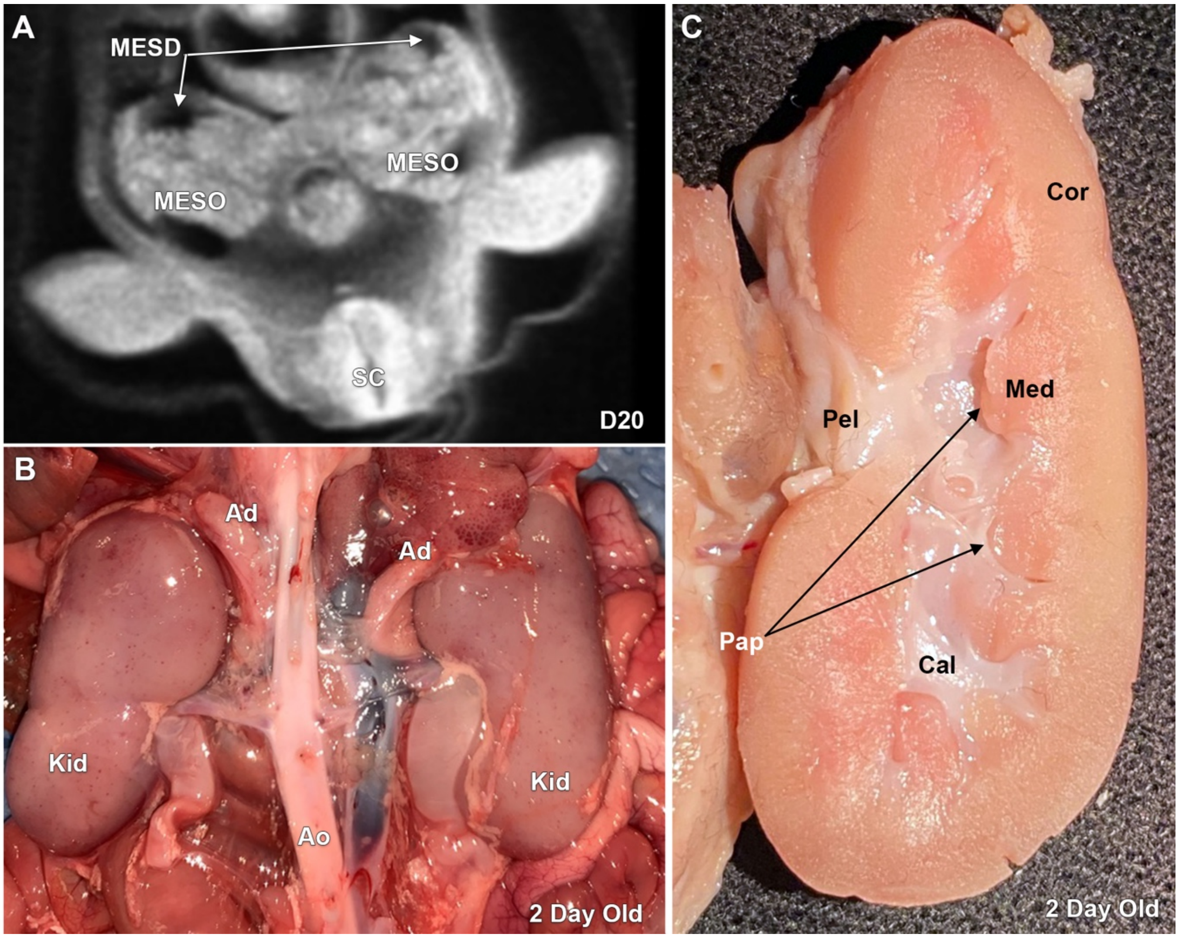

S2

Supplemental Figure 2. The mesonephric ducts are shown in a D20 GA pig in panel (A). (B) shows a posterior view (dorsal side of the pig) of the kidneys attached to the visceral block, and (C) the cut surface of a kidney in a 2-day old pig. MESD-Mesonephric duct, MESO-Mesonephrous, SC-Spinal cord, Kid-Kidney, Ad-Adrenal gland, Ao-Aorta, Cor-Renal cortex, Cal-Calyces, Med-Renal medulla, Pap-Renal papillae, Pel-renal Pelvis.

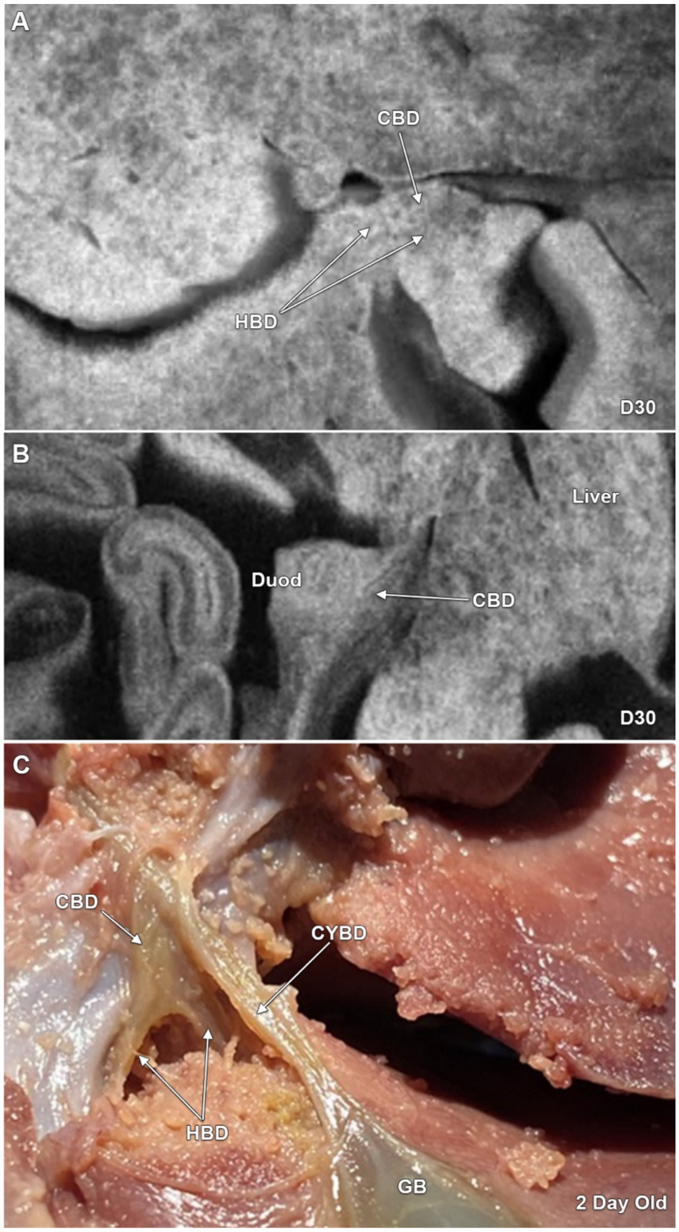

S3

Supplemental Figure 3. Cross-sections of the hepatic and common bile ducts are demonstrated in (A), and the common bile duct is seen extending to the duodenum in (B) in a D30 GA piglet. (C) is a gross dissection of the extrahepatic bile ducts in a 2-day old pig. CBD-Common bile duct, HBD-Hepatic bile duct, Duod-Duodenum. CYBD-Cystic bile duct, GB-Gallbladder.

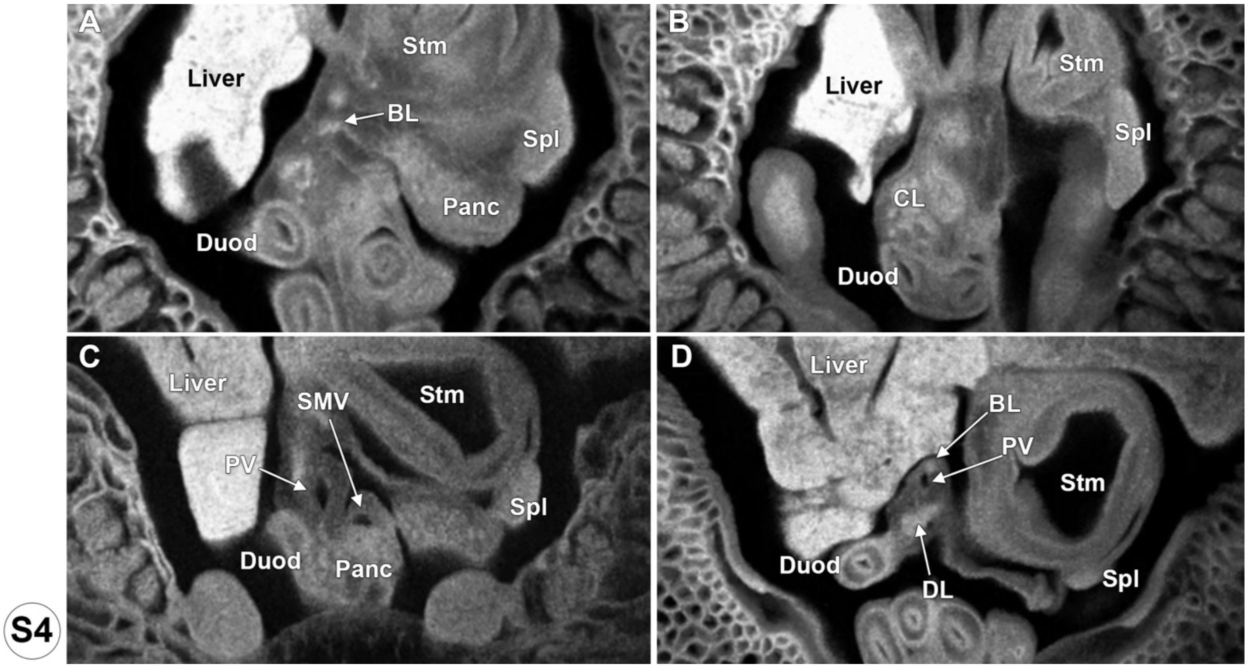

Supplemental Figure 4. ECM images of a pig fetus showing the structure of the pancreas at D30 GA. The pancreatic bridge and the splenic lobe of the pancreas are shown in (A), and (B) shows the connecting lobe of the pancreas. (C and D) illustrate the association between the pancreas and the portal vein. Stm-Stomach, Spl-Spleen, Panc-Pancreas, BL-Pancreatic bridge lobe, Duod-Duodenum, CL-Connecting lobe of the pancreas, PV-Portal vein, SMV-Superior mesenteric vein, DL-Duodenal lobe of the pancreas.

SUPPLEMENTAL VIDEO LEGENDS

Supplemental Video 1. ECM image stack reconstruction of the Day 20 pig embryo in the coronal plane.

Supplemental Video 2. ECM image stack reconstruction of the Day 20 pig embryo in the sagittal plane.

Supplemental Video 3. ECM image stack reconstruction of the Day 20 pig embryo in the transverse plane.

Supplemental Video 4. ECM image stack reconstruction of the Day 26 pig embryo abdomen in the coronal plane.

Supplemental Video 5. ECM image stack reconstruction of the Day 30 pig embryo abdomen in the coronal plane.

Supplemental Video 6. MRI image stack reconstruction of the Day 35 pig embryo abdomen in the coronal plane.

Supplemental Video 7. MRI image stack reconstruction of the Day 35 pig embryo abdomen in the sagittal plane.

Supplemental Video 8. MRI image stack reconstruction of the Day 35 pig embryo abdomen in the transverse plane.

Supplemental Video 9. MRI image stack reconstruction of the Day 42 pig embryo in the coronal plane.

Supplemental Video 10. MRI image stack reconstruction of the Day 42 pig embryo in the sagittal plane.

Supplemental Video 11. MRI image stack reconstruction of the Day 42 pig embryo in the transverse plane.

Supplemental Video 12. MRI image stack reconstruction in the coronal plane of the abdomen of a in *SAP130* mutant newborn pig.

Supplemental Video 13. MRI image stack reconstruction in the sagittal plane of the abdomen of a in *SAP130* mutant newborn pig.

Supplemental Video 14. MRI image stack reconstruction in the transverse plane of the abdomen of a in *SAP130* mutant newborn pig.

SUPPLEMENTAL REFERENCES

1 Zorn, A. Liver development (October 31, 2008), StemBook, ed. *The Stem Cell Research Community, StemBook, doi/10.3824/stembook* **1** (2008).

2 Hsu, C. W. *et al.* Three-dimensional microCT imaging of mouse development from early post-implantation to early postnatal stages. *Dev Biol* **419**, 229-236, doi:10.1016/j.ydbio.2016.09.011 (2016).

3 Davidson, A. J. Mouse kidney development. *International Journal of Biomedical and Health Sciences* **10** (2021).

4 McMahon, A. P. Development of the mammalian kidney. *Current topics in developmental biology* **117**, 31-64 (2016).

5 Seely, J. C. A brief review of kidney development, maturation, developmental abnormalities, and drug toxicity: juvenile animal relevancy. *Journal of toxicologic pathology* **30**, 125-133 (2017).

6 Ungewitter, E. & Yao, H.-C. How to make a gonad: cellular mechanisms governing formation of the testes and ovaries. *Sexual Development* **7**, 7-20 (2013).

7 Kim, J.-H. & Choi, M. H. Embryonic development and adult regeneration of the adrenal gland. *Endocrinology and Metabolism* **35**, 765 (2020).
